# VCISpain: protocol for a prospective multicenter observational study to validate a standardized classification tool for tracheal intubation using videolaryngoscopy

**DOI:** 10.1016/j.bjane.2025.844653

**Published:** 2025-06-18

**Authors:** Miguel Ángel Fernández-Vaquero, Pedro Charco-Mora, Jose Alfonso Sastre-Rincon, Manuel Ángel Gomez-Rios, Johanes Cardenas-Gomez, Eugenio Martinez-Hurtado, Norma Aracil-Escoda, Andrea Vallejo-Tarrat, Inés Thion-Soriano, David Peral-Sanchez, Miguel Castañeda-Pascual, Miguel Rodriguez del Rio, Nekari de Luis-Cabezon, Joseba Gonzalez-Garcia, Jesus Acosta-Martinez, Silvia Gonzalez-Santos, Xavier Onrubia-Fuertes, Estefania Martinez-Gonzalez, Andres Roca de Togores-Lopez, Iratxe Gonzalez-Mendibil, Almudena Baños-Maestro, Marisa Mariscal-Flores, Aitana Lluch-Oltra, Izaskun Emazabel-Yunta, Diana M. Rodriguez-Sanabria, José Manuel Álvarez-Avello, Diego A. Meléndez Salinas, Teresa Lopez-Correa, Miriam Sanchez-Merchante, Maria Bermudez-Lopez, Elena Martinez-Gallego, Maria C. Martinez-Segovia, Ana Belen Martin-Andres, Yaiza Molero-Diez, Miguel A. Garcia-Aroca, Blanca Tapia-Salinas, Jaume Puig-Bernabe, Andrea Piano, Gloria Hernandez-Fernandez, Alvaro Mingote-Lladó, Javier Garcia-Fernandez, Javier Mata-Estevez, Laura Reviriego-Agudo, Gabriel Ruiz-Cordoba, Javier Moya-Moradas, Francisco Javier Marques-Asin, Marc Vives Santacana, VCISpain Trial Investigators Group

**Affiliations:** a Department of Anesthesiology and Critical Care Clínica Universidad Navarra, Madrid, Spain; b Department of Anesthesiology and Critical Care Hospital Universitario y Politécnico la Fe, Valencia, Spain; c Department of Anesthesiology and Critical Care Complejo Asistencial Universitario de Salamanca, CAUSA, Salamanca, Spain; d Department of Anesthesiology and Critical Care Complejo Hospitalario Universitario de A Coruña, CHUAC, A Coruña, Spain; e Department of Anesthesiology and Critical Care Hospital Infanta Sofía, San Sebastián de los Reyes, Madrid, Spain; f Department of Anesthesiology and Critical Care Hospital Universitario Infanta Leonor, Madrid, Spain; g Department of Anesthesiology and Critical Care Hospital Clínic de Barcelona, Barcelona, Spain; h Department of Anesthesiology and Critical Care Hospital Sant Joan Despí Moises Broggi, Barcelona, Spain; i Department of Anesthesiology and Critical Care Consorcio Hospital General de Castellón, Spain; j Department of Anesthesiology and Critical Care H. Universitario de Navarra, Spain; k Department of Anesthesiology and Critical Care HM Hospitales, Madrid, Spain; l Department of Anesthesiology and Critical Care Hospital Universitario de Basurto, Bilbao, Spain; m Department of Anesthesiology and Critical Care H Universitario Virgen del Rocio, Sevilla, Spain; n Department of Anesthesiology and Critical Care H. U. Donostia, San Sebastián, Spain; o Department of Anesthesiology and Critical Care Hospital Universitari Dr. Peset, Valencia, Spain; p Department of Anesthesiology and Critical Care Hospital General Requena, Valencia, Spain; q Department of Anesthesiology and Critical Care Hospital de Galdakao-Usansolo, Galdakao, Spain; r Department of Anesthesiology and Critical Care Hospital Universitario de Getafe, Madrid, Spain; s Department of Anesthesiology and Critical Care Hospital del Bidasoa, Irún, Spain; t Department of Anesthesiology and Critical Care HUA, Vitoria; u Department of Anesthesiology and Critical Care Hospital Universitario Fundación Alcorcón, Madrid, Spain; v Department of Anesthesiology and Critical Care Hospital Universitario Lucus Augusti, Lugo, Spain; w Department of Anesthesiology and Critical Care H U Virgen Arrixaca, Murcia, Spain; x Department of Anesthesiology and Critical Care Complejo Asistencial Zamora, Spain; y Consultant at Department of Anesthesiology and Critical Care Hospital Central de la Defensa “Gomez Ulla”, Madrid, Spain; z Department of Anesthesiology and Critical Care H.U. La Paz, Madrid, Spain; aa Department of Anesthesiology and Critical Care Consorcio Hospital General Universitario Valencia, Spain; bb Department of Anesthesiology and Critical Care Hospital Universitario Torrecardenas, Almeria, Spain; cc Department of Anesthesiology and Critical Care Hospital Universitario Gregorio Marañon, Madrid, Spain; dd Department of Anesthesiology and Critical Care Hospital Universitario Puerta de Hierro, Madrid, Spain; ee Department of Anesthesiology and Critical Care Hospital Son Llatzer, Mallorca, Spain; ff Department of Anesthesiology and Critical Care Hospital Clinico de Valencia, Spain; gg Department of Anesthesiology and Critical Care Hospital FREMAP, Madrid, Spain; hh Department of Anesthesiology and Critical Care Hospital Universitario Ramón y Cajal, Madrid, Spain; ii Department of Anesthesiology and Critical Care Hospital Universitario Virgen Macarena, Sevilla, Spain; jj Consultant at Clinica at Department of Anesthesiology and Critical Universidad Navarra, Pamplona, Spain

**Keywords:** Airway management, Patient care, Reproducibility of results, Tracheal intubation, Video-assisted techniques and procedures

## Abstract

**Background and objective:**

Videolaryngoscopy have transformed airway management by improving intubation success rates compared to direct laryngoscopy. However, its widespread adoption has been hindered by the lack of standardized classification tools for documentation and communication. This manuscript outlines the rationale and study design of the VCISpain project, which aims to evaluate the interobserver reproducibility of the Video Classification of Intubation (VCI) scale in the context of airway management using videolaryngoscopy in Spain.

**Methods:**

This manuscript presents the planned methodology of the VCISpain study, a prospective, observational, multicenter, open-label study. The study will collect data on tracheal intubations performed in operating rooms, intensive care units, and emergency departments. In each case, two anesthesiologists will independently apply the VCI scale to assess blade type, Percentage of Glottic Opening (POGO), and ease of intubation.

**Ethics and registration:**

The study was approved by the University of Navarra Ethics Committee (2022.079 mod1) and registered on ClinicalTrials.gov (NCT06537531). It is endorsed by the Spanish Society of Anesthesiology, Resuscitation and Pain Therapy (SEDAR) and the European Airway Management Society (EAMS).

**Conclusions:**

VCISpain seeks to establish a standardized classification tool for documenting and communicating findings related to videolaryngoscopy in airway management. By presenting the study rationale and design, this protocol aims to promote transparency, ensure methodological rigor, and encourage broader discussion to refine the study prior to implementation.

## Introduction

Tracheal Intubation (TI) remains a cornerstone of airway management in both anesthesia and critical care, despite its routine use.^1^^,^^2^ Difficult intubation occurs in approximately 5%–8% of cases, while failed TI is reported in 0.05%–0.35% of cases.^3^ Recent data indicate a decline in these rates ‒ 1.6 and 0.06 per 1,000 cases, respectively ‒ primarily due to advances such as Videolaryngoscopy (VL).^4^ Nonetheless, airway management complications remain a significant cause of morbidity and mortality.^5^^,^^6^

Direct Laryngoscopy (DL) has traditionally been the gold standard for visualizing the glottis and guiding tracheal tube placement.^7^ The advent of VL in 2001 marked a significant milestone, reducing rates of difficult and failed airways.^8^ Consequently, several national and international societies ‒ including the American Society of Anesthesiologists (ASA), the Canadian Airway Focus Group (CAFG), and the Spanish Society of Anesthesiology and Resuscitation (SEDAR) ‒ now recommend VL as a first-line technique for TI due to its ability to reduce complications and improve clinical outcomes.^9^, ^10^, ^11^

Traditional classification tools ‒ such as the Cormack-Lehane scale, its modification by Yentis and Cook, and the Percentage of Glottic Opening (POGO) ‒ remain the most commonly used methods for assessing intubation difficulty.^7^^,^^12^, ^13^, ^14^ However, these tools were originally developed for direct laryngoscopy and may not fully capture the specific challenges associated with VL.^15^ For example, excellent glottic visualization (e.g., 100% POGO) during VL does not always guarantee procedural success^16^^,^^17^ due to difficulties in maneuvering and inserting the tube through the glottis.^18^, ^19^, ^20^ Although alternative scales have been proposed to overcome these limitations, none have gained widespread acceptance.^16^^,^^21^

The Video Classification of Intubation (VCI) scale was developed to address this gap, providing a standardized classification tool for documenting VL intubations. It evaluates three components: blade type (Macintosh [M] or Hyperangulated [H]), Percentage of Glottic Opening (POGO), and ease of intubation (Easy [E], Difficult [D], or Failed [F]).^22^ The VCISpain study is a national, multicenter initiative aimed at evaluating the interobserver reproducibility and clinical applicability of the VCI scale in real-world practice across Spain. The study is endorsed by the Spanish Society of Anesthesiology, Resuscitation, and Pain Therapy (SEDAR) and the European Airway Management Society (EAMS). This manuscript presents the study rationale and design to promote transparency, ensure methodological rigor, and encourage constructive feedback prior to implementation.^23^

## Methods

### Objectives

#### Primary Aim

The primary aim of this study is to evaluate the interobserver reproducibility of the VCI scale during tracheal intubation using videolaryngoscopy across multiple centers in Spain.

#### Secondary aims

1. To assess the correlation between the Percentage of Glottic Opening (POGO) and the difficulty of tracheal intubation.
2. To evaluate the impact of the operator’s experience level on intubation-related outcomes.
3. To determine the incidence of complications associated with tracheal intubation using videolaryngoscopy.

### Study design

The VCISpain study is a prospective, observational, multicenter, and open-label study. This design enables data collection across a diverse range of hospital settings, enhancing the external validity and generalizability of the findings. This protocol was developed in accordance with the STROBE (Strengthening the Reporting of Observational Studies in Epidemiology) guidelines for observational studies.

### Study setting

The project involves 35 hospitals located across various autonomous communities in Spain. These centers encompass a wide array of clinical contexts, levels of care complexity, and technological capabilities, thereby ensuring a heterogeneous and representative sample. Additionally, the study includes anesthesiologists with varying levels of experience, offering a comprehensive and realistic analysis of the VCI scale’s application in clinical practice.

The list of participating hospitals is as follows:

1. Clínica Universidad de Navarra (Madrid).
2. Clínica Universidad de Navarra (Pamplona).
3. Complejo Asistencial Universitario de Salamanca (Salamanca).
4. Complejo Hospitalario Universitario de A Coruña (A Coruña).
5. Complejo Asistencial de Zamora (Zamora).
6. Consorcio Hospital General de Castellón (Castellón).
7. Hospital Clínic de Barcelona (Barcelona).
8. Hospital del Bidasoa (Irún).
9. Hospital de Galdakao-Usansolo (Galdakao).
10. Hospital General de Requena (Valencia).
11. Hospital Infanta Sofía (Madrid).
12. Hospital Sant Joan Despí (Barcelona).
13. Hospital Universitari Dr. Peset (Valencia).
14. Hospital Universitario de Álava (HUA-Vitoria).
15. Hospital Universitario de Basurto (Bilbao).
16. Hospital Universitario de Getafe (Madrid).
17. Hospital Universitario de Navarra (Pamplona).
18. Hospital Universitario Donostia (San Sebastián).
19. Hospital Universitario Fundación Alcorcón (Madrid).
20. Hospital Universitario Infanta Leonor (Madrid).
21. Hospital Universitario La Fe (Valencia).
22. Hospital Universitario Lucus Augusti (Lugo).
23. Hospital Universitario Virgen de la Arrixaca (Murcia).
24. Hospital Universitario Virgen del Rocío (Sevilla).
25. HM Hospitales (Madrid).
26. Hospital Universitario La Paz (Madrid).
27. Consorcio Hospital General Universitario Valencia.
28. Hospital Universitario Torrecárdenas (Almería).
29. Hospital Universitario Gregorio Marañón (Madrid).
30. Hospital Universitario Puerta de Hierro (Madrid).
31. Hospital Son Llátzer (Mallorca).
32. Hospital Clinico de Valencia.
33. Hospital FREMAP (Madrid).
34. Hospital Universitario Ramón y Cajal (Madrid).
35. Hospital Universitario Virgen Macarena (Sevilla).

This extensive network of hospitals ensures a comprehensive representation of the variability and complexity encountered in airway management across Spain.

### Pre-data collection preparations

To ensure consistent and standardized application of the VCI scale across all centers, a comprehensive training and coordination strategy was implemented before the initiation of data collection. All participating anesthesiologists completed a structured, one-hour virtual training session led by the principal investigator (MFV) and study coordinators (PC, MV). The session included a detailed review of the study protocol, proper completion of the Case Report Form (CRF), and specific guidance on applying the VCI scale. Emphasis was placed on protocol adherence, data quality, and ethical considerations.

Each participating hospital received a complete study package containing the CRF, informed consent templates, and all necessary supporting documentation for protocol implementation. To ensure ongoing support, a dedicated online discussion forum was created to allow investigators to submit questions and receive timely clarifications. In addition, monthly virtual meetings are being held with all site investigators to reinforce protocol adherence, resolve methodological concerns, and ensure consistency in the interpretation and documentation of the VCI scale across centers.

All participating centers obtained approval from their ethics committees, ensuring compliance with applicable ethical and regulatory standards throughout the study.

### Eligibility criteria

This study will include adult patients (≥ 18-years-old) with an American Society of Anesthesiologists (ASA) physical status classification of I to III who require TI in a variety of clinical contexts, including diagnostic, therapeutic, or surgical procedures, as well as airway management in the operating room, Post-Anesthesia Care Unit (PACU), Intensive Care Unit (ICU), or emergency department. Eligible patients must undergo TI performed by an anesthesiologist or anesthesia resident participating in the study, and written informed consent must be obtained from the patient or their legal representative prior to the procedure.

### Intervention in the VCISpain study

The intervention begins during the pre-anesthetic consultation or in the anesthetic-surgical preparation area (preoperative holding area), where eligible patients are provided with a detailed information sheet outlining the study objectives, methodology, and potential risks. The investigator anesthesiologists explain the protocol, address any questions or concerns, and ensure patient understanding before obtaining written informed consent.^24^ Participants are informed of their right to withdraw consent at any time. If consent is withdrawn, the participant’s data will be excluded from analysis in accordance with ethical guidelines, thereby respecting their autonomy (Appendices 1–2).

**Figure 1:**
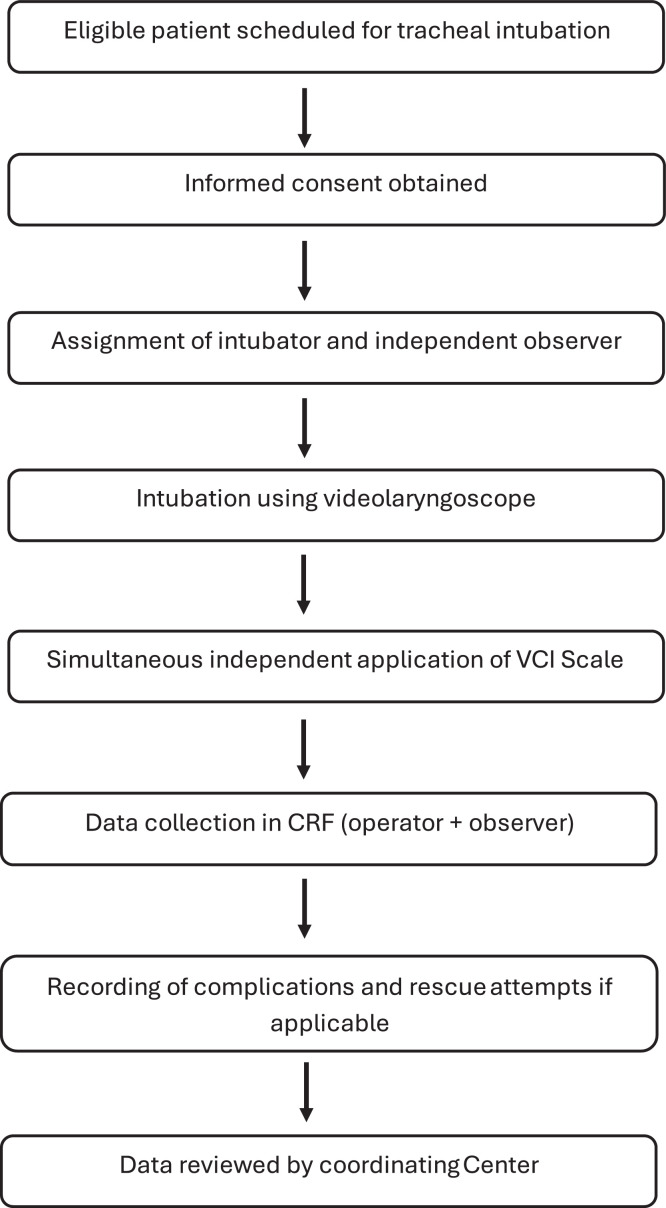
Flowchart.

**Figure 2:**
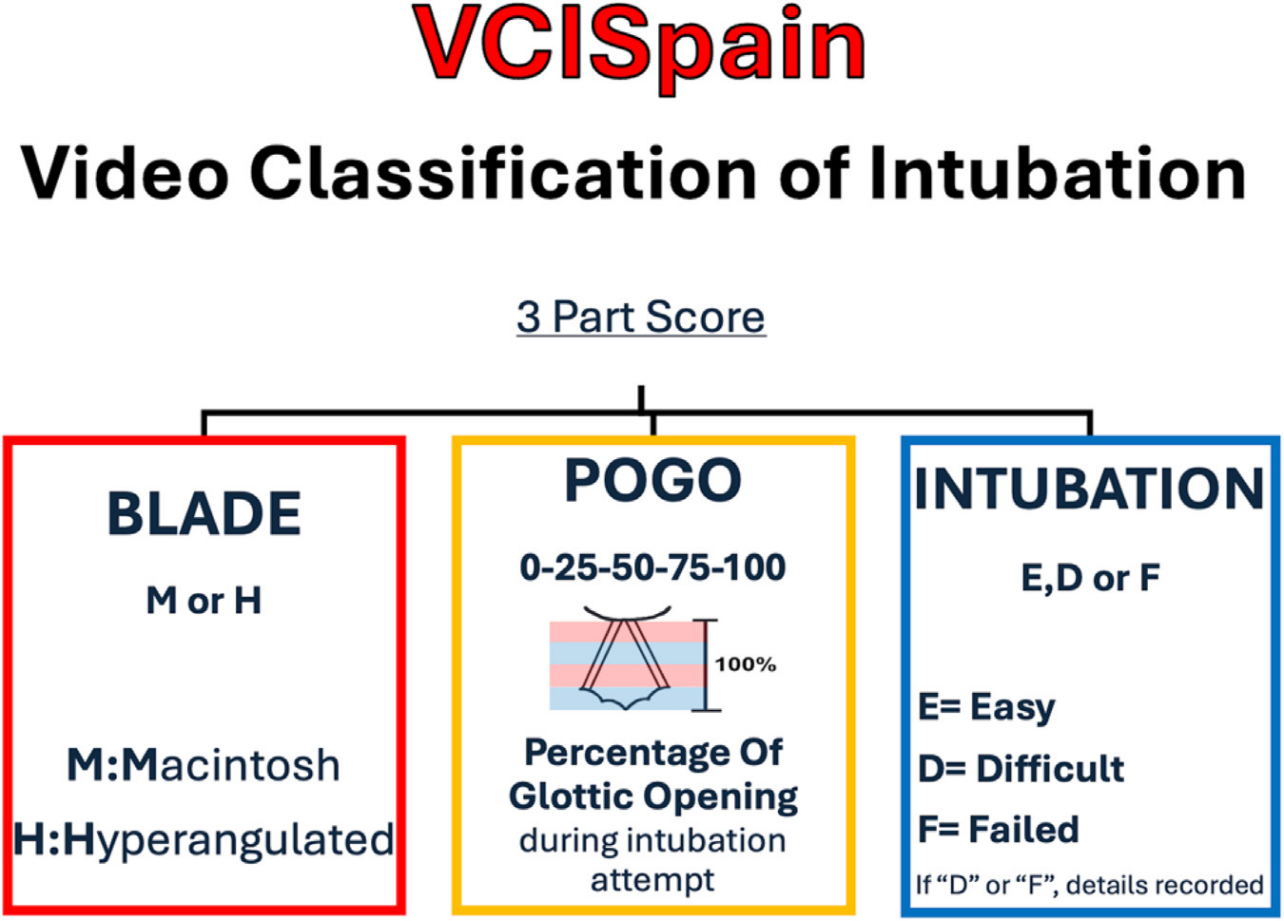
VCISpain (Video Classification of Intubation).

The clinical procedure follows standard practices (Fig. 1). TI is performed using a videolaryngoscope selected based on patient characteristics and the resources available at each center. The responsible anesthesiologist evaluates the TI using the VCI scale, which comprises the following components (Fig. 2):^22^

1. Blade Type: The videolaryngoscope is classified as either Macintosh (M) or hyperangulated (H).
2. Percentage of Glottic Opening (POGO): The POGO score is recorded at a standardized time point ‒ immediately before advancing the endotracheal tube into the glottis. This measurement reflects the actual glottic view under the force and positioning used during intubation. Investigators are instructed not to record the initial or best view, but rather the view observed at the moment of tube insertion, to ensure consistency and clinical relevance.
3. Ease of Intubation: This is categorized as easy (E), difficult (D), or failed (F).
  a) Easy refers to successful tracheal intubation using the manufacturer’s standard technique for the selected videolaryngoscope, without the need for adjuncts or external assistance.
  b) Difficult is defined as intubation requiring the use of adjuncts such as a bougie, stylet, or other guiding devices to facilitate tube placement.
  c) Failed refers to the inability to intubate the trachea using the initially selected videolaryngoscope, necessitating the use of a different videolaryngoscope or an alternative device (e.g., fiberoptic bronchoscope, supraglottic airway, or surgical airway).

Simultaneously, a second anesthesiologist observes the tracheal intubation procedure and independently records the VCI score to assess interobserver reproducibility.

Complications will be prospectively recorded based on predefined clinical criteria, including:^25^ Hypoxemia (SpO_2_ < 90% for ≥ 10 seconds); Esophageal intubation (confirmed by capnography or clinical signs); Dental injury (visible tooth damage); Laryngeal trauma (presence of blood on the blade or tube, hoarseness, or stridor); Bronchospasm or laryngospasm (clinically diagnosed); Failed intubation (requiring a change in device or technique).

If a serious adverse event related to the videolaryngoscope is suspected, the attending physician may temporarily suspend the patient’s participation in the study. Throughout the process, patient safety is prioritized, and each center's standard protocols will be followed in the event of any incident.

### Outcome measures

#### Primary outcome

The primary outcome is the inter-rater agreement and reproducibility of the VCI scale, defined as the level of concordance between the VCI scores assigned by the anesthesiologist performing the tracheal intubation and an independent observer.

#### Secondary outcomes

1. The correlation between the Percentage of Glottic Opening (POGO) score and the difficulty of tracheal intubation.
2. The impact of operator experience on VCI scale outcomes, including the influence of training level and prior videolaryngoscopy experience on interobserver agreement and VCI scoring.
3. The incidence of tracheal intubation–related complications. While complications such as hypoxemia and laryngeal trauma are included as secondary outcomes, their expected low frequency means that related analyses will remain exploratory and descriptive in nature.

### Exploratory data

The study will collect essential data in the following domains (Table 1) (Appendix 3):

1) Patient Demographics: Includes age, sex, weight, height, Body Mass Index (BMI), and ASA status.
2) Operator Characteristics: Documents the experience of the intubator or observer categorized by role (resident or specialist), years of experience (< 4, 4–8, > 8), and the number of prior intubations with VL: < 25, 25–50, > 50.
3) Procedure Setting: Documents the clinical environment, including operating theater, ICU, Post-Anesthesia Care Unit (PACU), or emergency department.
4) Videolaryngoscope Characteristics: Captures the type of videolaryngoscope used during the procedure.
5) Data Related to the VCI Scale: a) VCI score: Includes blade type (Macintosh or hyperangulated), POGO (categorized as < 25%, 25%–50%, 50%–75%, > 75%), and difficulty (easy, difficult, failed); b) Rescue Devices: If a rescue device is used, a new VCI score will be recorded.
6) Complications: these include hypoxemia, esophageal intubation, dental injury, laryngeal trauma, bronchospasm or laryngospasm, and failed intubation.

**Table 1:** CRF VCISpain. VIDEO CLASSIFICATION OF INTUBATION VCISpain – Case Report Form (CRF).

**General Information**	
Date	
Intubator’s e-mail	
Patient Demographics	
Age	
Sex/Gender	
ASA status	
Weight (kg)	
Height (cm)	
**Intubation Setting**	
Place of Intubation	Operating Room / ICU / Emergency / Other
**Videolaryngoscope Details**	
Videolaryngoscope Model	McGrath / C-MAC / Airtraq / Glidescope / Others
**Intubator Data**	
Role	Resident / Specialist
Years of experience	< 4 / 4–8 / > 8
Prior VL intubations	< 25 / 25–50 / > 50
Intubator VCI	
Blade type	Macintosh / Hyperangulated
POGO score	< 25% / 25–50% / 50–75% / > 75%
Ease of intubation	Easy / Difficult / Failed
If Difficult	Stylet / Bougie / Other adjunct
If Failed	Rescue device used + Rescue VCI
**Observer Data**	
Role	Resident / Specialist
Years of experience	< 4 / 4–8 / > 8
Prior VL intubations	< 25 / 25–50 / > 50
**Observer VCI**	
Blade type	Macintosh / Hyperangulated
POGO score	< 25% / 25–50% / 50–75% / > 75%
Ease of intubation	Easy / Difficult / Failed
If Difficult	Stylet / Bougie / Other adjunct
If Failed	Rescue device used + Rescue VCI
**Complications**	
Desaturation	< 92%
Esophageal intubation	Yes / No
Dental damage	Yes / No
Other	Specify
**Rescue VCI (If applicable)**	
Blade type	Macintosh / Hyperangulated
POGO score	< 25% / 25–50% / 50–75% / > 75%
Ease of intubation	Easy / Difficult / Failed

### Data collection and management

Data will be collected using a standardized and anonymized physical CRF, independently completed by both the intubating anesthesiologist and the observer during and immediately after the intubation procedure. The collected data will then be managed through the secure electronic platform Research Electronic Data Capture (REDCap), hosted at the University of Navarra (UNAV), ensuring stringent patient confidentiality and data integrity. REDCap offers validated data capture and a transparent audit trail through its comprehensive logging features.

### Data management workflow

Initial Data Capture: after each procedure, the anesthesiologist will complete the CRF, recording demographic data, procedure variables, and any observed complications.

• Data Transfer to REDCap: the pseudo-anonymized data will be entered into the secure REDCap platform. Each patient will be assigned a unique encoding code that is not directly linked to their personal information, ensuring confidentiality.
• Ethical and Regulatory Compliance: all data will be collected and stored in full compliance with Good Clinical Practice (GCP) guidelines and current data protection regulations.

### Data protection and quality assurance

Data Protection: the database will be encrypted and accessible only via individual passwords assigned to each investigator, ensuring robust security.

• Protocol Supervision: a designated clinical investigator will oversee the implementation of the study protocol, thoroughly documenting any deviations, adverse events, or protocol violations.
• Audits: the coordinating team and the principal investigator will conduct regular audits to verify data integrity and quality, maintaining high research standards.

### Data access and oversight

• Centralized Access: the principal investigator (MFV) and two coordinators (PC and MV) will manage centralized access to the data, ensuring supervision of data security, quality, and statistical analysis.
• Access for Participating Centers: data will be made available to all participating centers to promote transparency and foster collaboration during analysis.
• Centralized Oversight: the principal investigator will coordinate the processes of data collection, storage, and analysis.

### Statistical methods

A comprehensive statistical analysis will be conducted to describe the study’s quantitative and qualitative variables. Quantitative variables will be summarized using measures of central tendency and dispersion (mean ± standard deviation or median [interquartile range]), depending on the distribution of data. Qualitative variables will be presented as absolute frequencies and percentages.

To evaluate the interobserver reproducibility of the VCI scale among anesthesiologists, the Cohen’s Kappa coefficient will be used to measure the level of agreement between the anesthesiologist performing the tracheal intubation and the independent observer. Interpretation of agreement will follow standard criteria (≤ 0.20 poor, 0.21–0.40 fair, 0.41–0.60 moderate, 0.61–0.80 substantial, and > 0.80 almost perfect).

Binary logistic regression analysis will be performed to explore associations between key variables, specifically to assess the relationship between the Percentage of Glottic Opening (POGO) and the ease or difficulty of Tracheal Intubation (TI). Results will be reported as Odds Ratios (OR) with their corresponding 95% Confidence Intervals (95% CI).

Statistical significance will be determined using a p-value threshold of < 0.05. Data analyses will be performed using Stata 18 (StataCorp, College Station, TX, USA), a software providing advanced modeling and data-processing capabilities to ensure the precision and validity of the results.

### Sample size

The study will enroll a total of 1,395 patients, accounting for a 5% dropout rate. The sample size calculation is based on achieving a precise estimate of the expected interobserver agreement measured by Cohen’s Kappa coefficient, assumed to be 0.80, with a desired precision of ± 0.11 and a confidence interval of 95%. Additionally, assuming a 10% incidence of difficult Tracheal Intubation (TI), this sample size will ensure adequate representation for meaningful secondary analyses.^6^ Patient recruitment is anticipated to take between 12 and 18 months, starting in September 2024.

### Ethics and dissemination

#### Ethical approval of research

The VCISpain study complies with the principles outlined in the Declaration of Helsinki and the GCP Guidelines. Ethical approval has been granted by the Research Ethics Committee of the University of Navarra (session of September 5, 2024, reference 2022.079 mod1). The study was registered at ClinicalTrials.gov (NCT06537531), ensuring adherence to transparency and high ethical standards. Oversight of ethical compliance will be managed by the principal investigator and coordinators in collaboration with the University of Navarra (UNAV).

#### Confidentiality

To uphold participant confidentiality, all original records will be securely stored at the participating centers for five years after the study’s completion. The electronic database will be thoroughly cleaned, anonymized, and retained for this period; this approach guarantees compliance with data protection regulations and ensures the safeguarding of participant information.

## Discussion

Airway management is a cornerstone of anesthetic practice; however, difficult tracheal intubation remains a significant concern, as highlighted by a recent audit in the United Kingdom.^6^ The introduction of VL has transformed airway management by providing superior glottic visualization, increasing first-attempt success rates, and reducing complications associated with multiple intubation attempts, such as hypoxemia, laryngeal trauma, and esophageal intubation.^25^ Nevertheless, the widespread adoption of VL continues to be limited by challenges related to training,^26^ financial constraints, and, notably, the lack of a standardized classification tool for documenting and communicating VL findings.^23^

The VCISpain study aims to bridge this gap by validating the VCI scale. This tool is designed to establish a standardized and reproducible language for airway management with videolaryngoscopy, addressing the limitations of traditional classification systems such as the Cormack-Lehane scale. Unlike these conventional tools, the VCI scale captures the unique features of videolaryngoscopy, particularly the “you see, and you fail” phenomenon, in which excellent glottic visualization does not necessarily ensure successful intubation.^17^ By standardizing communication, the VCI scale can enhance planning for future airway treatments, ultimately promoting patient safety.

The VCI scale integrates three key components: blade type (Macintosh or hyperangulated), the POGO score, and the ease or difficulty of the intubation procedure. This tool not only enhances documentation but also facilitates communication among clinicians, while supporting training and standardization in advanced airway management.

A previous study demonstrated the accuracy and reproducibility of the VCI scale in describing VL intubations.^22^ With its multicenter design ‒ encompassing 35 Spanish hospitals and anesthesiologists with varying levels of experience ‒ the VCISpain study provides a representative reflection of real-world clinical practice in Spain. Moreover, standardized training on the study protocol and the use of the VCI scale will help ensure the quality and reproducibility of the collected data.

This study offers several strengths, including the pioneering evaluation of the VCI scale in a multicenter setting, providing robust data on its reproducibility and clinical utility. Its pragmatic design minimizes interference with standard care, enhancing its relevance to everyday clinical practice.

Despite certain limitations ‒ such as heterogeneity among participating centers and variability in videolaryngoscope models and operator experience ‒ these factors may, in fact, increase the external validity of the findings by reflecting real-world clinical diversity.

One notable limitation is the absence of a direct comparison with existing classification systems, which prevents definitive conclusions regarding the superiority of the VCI scale over other tools.^16^ However, although not directly compared in this study, the VCI scale may offer advantages by integrating blade type, glottic view, and ease of intubation into a single, videolaryngoscopy-specific tool. It enhances clinical communication and documentation, not by replacing traditional scales, but by complementing them with context-specific information relevant to modern airway management.

The VCISpain study represents a significant step toward standardizing the communication of information related to videolaryngoscopy-guided tracheal intubation.^27^ Validation of the VCI scale has the potential to establish a new benchmark for both national and international clinical practice, informing future airway management guidelines. Integrating the VCI scale into routine care may improve patient safety, foster interdisciplinary collaboration, and support continuous professional development.

## Conclusion

In conclusion, VCISpain represents a cultural shift in airway management through the use of videolaryngoscopy. By establishing a common language, it has the potential to promote interprofessional collaboration, support clinical education, and improve airway management planning. It also paves the way for future research in anesthesiology.

Beyond its practical applications, the VCI scale reflects a broader commitment to continuous improvement and innovation in patient care, emphasizing safety and effective clinical communication as cornerstones of modern anesthetic practice.

## Conflicts of interest

The authors declare no conflicts of interest.

## Appendix Supplementary materials

Image, application 1

## Footnotes

Supplementary material associated with this article can be found in the online version at doi:10.1016/j.bjane.2025.844653.
