# Supplementary material for: VCISpain: protocol for a prospective multicenter observational study to validate a standardized classification tool for tracheal intubation using videolaryngoscopy
Source: Braz J Anesthesiol. 2025 Jun 18;75(5):844653. doi: 10.1016/j.bjane.2025.844653 (PMC12275188; doi:10.1016/j.bjane.2025.844653)
Supplement: Supplementary file 1 [file mmc1.docx]

BJAN-D-25-00032_Supplementary Material

**Appendix 1** Information Sheet.

| **INFORMATION SHEET:** |
| --- |
| Name of Coordinating Investigator: XXXXXXXXXXXXX, Clínica Universidad de Navarra. |
| Name of Investigator at the Center: |
| Research Center: |
| This information sheet explains the following: |
| **1. Invitation to Participate:** |
| You are invited to voluntarily participate, at no cost to you, in a research study titled “Video Classification of Intubation (VCI).” Please read this information carefully and ask any questions you may have before signing the informed consent form. Take your time, and if you wish, you may discuss it with a family member or close associate. |
| **2. Study Conduct:** |
| The study will be carried out by the local investigator at your center and coordinated by Dr. XXXXXXXXXXXXXXXX, the principal investigator overseeing all centers. He is a specialist in Anesthesiology and Resuscitation from the Department of Anesthesia at the Clínica Universidad de Navarra, with ID number XXXXXXX and email address XXXXXXXX. The informed consent form will be provided to you in advance during the pre-anesthesia consultation, allowing you sufficient time to address any questions you may have. |
| **3. Purpose of the Study:** |
| The purpose of this study is to evaluate the ease of use and reproducibility of a technique necessary for the general anesthesia procedure to which you will be subjected. This involves tracheal intubation, a routine procedure in our specialty. Intubation will be performed using a videolaryngoscope (a device inserted into the mouth equipped with a camera to visualize the vocal cords, through which a tube is placed to connect you to a ventilator to maintain lung function throughout the surgical procedure). The procedure will be conducted by an experienced specialist in Anesthesiology and Resuscitation.  No collection of specially protected data is required, and all information will be handled exclusively by the medical staff involved in the study. |
| **4. Voluntary Participation:** |
| As stated initially, your participation is entirely voluntary, and you may withdraw at any time without any adverse consequences. Should you decide to withdraw, you need to inform the project coordinator, Dr. XXXXXXXXXX. Your decision will not affect your medical treatment in any way. |
| **5. Personal Data Protection:** |
| All personal and clinical data will be processed in compliance with current data protection laws, particularly the GDPR and the Spanish Data Protection Act (LOPD).  The data controller, Clínica Universidad de Navarra (CUN), in compliance with Regulation (EU) 2016/679 of the European Parliament and of the Council of April 27, 2016 (GDPR), informs you that if you participate in this study, your clinical data will be processed by the research team to draw conclusions from the project. Health authorities and ethics committee members may also access your data if deemed necessary. Your identity will remain confidential in any study-related communications.  You are responsible for the accuracy of the data you provide and have the right to exercise access, rectification, deletion, limitation of processing, portability, and opposition of your data in accordance with data protection regulations. To do so, you may contact the Data Protection Officer at CUN by postal mail at XXXXXXXXXXX, by email at XXXXXXXXX, or through the website. Please include a copy of your national identity document or equivalent.  If you disagree with how your data is processed or believe your rights have been violated, you have the right to file a complaint with the Spanish Data Protection Agency. |
| **6. Confidentiality:** |
| Your medical records may be accessed by the local investigator at your center, and the necessary data for the study will be transferred exclusively to the coordinating investigator. |

**Appendix 2** Informed Consent.

**Appendix 3** Tables Variables.

| **Variable** | **Type** | **Definition / Scale** | **Time Point** | **Source** |
| --- | --- | --- | --- | --- |
| Date | Date | Date of procedure | Before procedure | Operator |
| Intubator’s e-mail | Text | Contact email of primary intubator | Before procedure | Operator |
| Age | Continuous | Age in years | Before procedure | Operator |
| Sex/Gender | Categorical | Male / Female / Other | Before procedure | Operator |
| ASA status | Ordinal | ASA physical status classification I–V | Before procedure | Operator |
| Weight | Continuous | Weight in kilograms | Before procedure | Operator |
| Height | Continuous | Height in centimeters | Before procedure | Operator |
| Place of intubation | Categorical | OR / ICU / Emergency / Other | Before procedure | Operator |
| Videolaryngoscope model | Categorical | McGrath / C-MAC / Airtraq / Glidescope / etc. | Before procedure | Operator |
| Role of intubator | Categorical | Resident / Specialist | Before procedure | Operator |
| Years of experience | Ordinal | < 4 / 4–8 / > 8 years | Before procedure | Operator |
| Prior VL intubations | Ordinal | < 25 / 25–50 / > 50 intubations | Before procedure | Operator |
| Blade type (operator) | Categorical | Macintosh / Hyperangulated | During intubation | Operator |
| POGO score (operator) | Ordinal | < 25%, 25–50%, 50–75%, > 75% | During intubation | Operator |
| Ease of intubation (operator) | Categorical | Easy / Difficult / Failed | During intubation | Operator |
| Adjuvants used (if difficult) | Categorical | Stylet / Bougie / External aid | During intubation | Operator |
| Role of observer | Categorical | Resident / Specialist | During intubation | Observer |
| Years of experience (observer) | Ordinal | < 4 / 4–8 / > 8 years | During intubation | Observer |
| Prior VL intubations (observer) | Ordinal | < 25 / 25–50 / > 50 intubations | During intubation | Observer |
| Blade type (observer) | Categorical | Macintosh / Hyperangulated | During intubation | Observer |
| POGO score (observer) | Ordinal | < 25%, 25–50%, 50–75%, > 75% | During intubation | Observer |
| Ease of intubation (observer) | Categorical | Easy / Difficult / Failed | During intubation | Observer |
| Complications | Categorical | Desaturation / Esophageal intubation / Dental damage / Other | During/after intubation | Operator |
| Rescue device used | Categorical | Brand/model of alternative device | If initial attempt fails | Operator |
| VCI (rescue) - blade type | Categorical | Macintosh / Hyperangulated | During rescue | Operator & Observer |
| VCI (rescue) ‒ POGO | Ordinal | < 25%, 25–50%, 50–75%, > 75% | During rescue | Operator & Observer |
| VCI (rescue) ‒ difficulty | Categorical | Easy / Difficult / Failed | During rescue | Operator & Observer |
